# Repositioning the Digital Rectal Examination in the Era of Risk-Adapted Prostate Cancer Diagnostics: Right Tool to the Right Patient at the Right Time

**DOI:** 10.1590/S1677-5538.IBJU.2026.9907

**Published:** 2026-03-30

**Authors:** Leonardo O. Reis

**Affiliations:** 1 Instituto Nacional de Ciência Tecnologia e Inovação em Câncer Geniturinário - INCT UroGen Campinas SP Brasil Instituto Nacional de Ciência, Tecnologia e Inovação em Câncer Geniturinário - INCT UroGen, Campinas, SP, Brasil; 2 Universidade Estadual de Campinas - Unicamp UroScience Faculdade de Ciências Médicas Campinas SP Brasil UroScience, Faculdade de Ciências Médicas, Universidade Estadual de Campinas - Unicamp, Campinas, SP, Brasil; 3 Instituto de Imuno-Oncologia Pontifícia Universidade Católica de Campinas - PUC-Campinas Escola de Ciências da Vida Campinas SP Brasil Instituto de Imuno-Oncologia, Escola de Ciências da Vida, Pontifícia Universidade Católica de Campinas - PUC-Campinas, Campinas, SP, Brasil

## COMMENT

Digital rectal examination (DRE) has long been a fundamental component of urological practice. Its attributes: low cost, safety, immediacy, and the ability to detect both prostate abnormalities and extra-prostatic findings, have historically justified its widespread use. However, the role of DRE in prostate cancer (PCa) detection has been substantially redefined, reflecting a broader shift toward precision-based, risk-adapted diagnostic pathways.

In the pre–multiparametric Magnetic Resonance Imaging (mpMRI) era, DRE played a more prominent role in the early detection of PCa. Approximately one-fifth of prostate cancers were identified solely based on abnormal DRE findings, and these tumors were often associated with higher Gleason grades and larger tumor volumes (1, 2). This observation suggested that DRE may have been inherently biased toward detecting clinically significant and biologically aggressive disease. Nonetheless, even in that context, its sensitivity remained limited, and its diagnostic contribution was highly dependent on patient characteristics and examiner experience.

The contemporary diagnostic landscape has evolved dramatically. Prostate-specific antigen (PSA) testing, combined with mpMRI and targeted biopsy strategies, now constitutes the backbone of early detection. These modalities offer superior sensitivity, improved risk stratification, and a more favorable balance between detection of clinically significant prostate cancer (csPCa) and avoidance of overdiagnosis. Consequently, DRE has progressively lost its role as a primary screening tool, especially in population screening.

This shift is now formally reflected in the 2026 updates of both the American Urological Association (AUA) and the European Association of Urology (EAU) guidelines, which no longer recommend DRE for routine screening in asymptomatic men. Instead, screening strategies are anchored in PSA-based risk assessment, with mpMRI subsequently used to guide biopsy decisions. This transition is supported by robust evidence demonstrating the limited sensitivity and reproducibility of DRE, as well as its contribution to false-positive referrals and unnecessary biopsies (3, 4).

A key limitation of DRE is its inherently subjective, operator-dependent nature. Interobserver variability is substantial, particularly among less experienced clinicians, leading to inconsistent diagnostic performance. Moreover, DRE lacks the ability to reliably detect anterior, small-volume, or early-stage tumors, which are often better visualized with mpMRI. These shortcomings directly impact screening efficiency and contribute to both over- and under-diagnosis (5, 6).

From a patient-centered perspective, DRE also represents a recognized barrier to screening participation. Reluctance to undergo the examination has been consistently reported and may reduce adherence to early detection programs. Equally important, a "normal" DRE may provide false reassurance, potentially delaying further investigation in men who would otherwise benefit from PSA testing and imaging (7).

Despite these limitations, it would be a misinterpretation to consider DRE obsolete. Rather, its role has become more selective and context-dependent. When used judiciously, DRE continues to provide clinically meaningful information that complements modern diagnostic tools. One such role is in estimating prostate volume (8), which enables the ready calculation of PSA density (PSAD). PSAD has emerged as a critical parameter in risk stratification to determine which patients need further evaluation with mpMRI. In this context, DRE, although less precise than imaging, can offer a rapid, bedside approximation of prostate volume that informs clinical decision-making, especially in resource-constrained environments. Also, in men with equivocal mpMRI findings, the PSAD helps to identify patients with a higher chance of benefit from a prostate biopsy.

Furthermore, DRE retains value in identifying a small but clinically relevant subset of prostate cancers that may be missed by PSA- and MRI-driven pathways, informing biopsy strategies in selected cases, especially when physical findings and imaging results do not align. These cases, although uncommon, are often characterized by higher-grade disease and larger tumor burden. Such observations reinforce the importance of maintaining clinical judgment alongside algorithm-based approaches (9).

From a diagnostic accuracy standpoint, DRE is best understood as a test with low sensitivity but relatively high specificity when findings are clearly abnormal. A distinctly suspicious DRE, such as nodularity, induration, or asymmetry, remains a strong indication for further investigation, regardless of PSA levels or imaging results. This characteristic supports its continued use in targeted clinical scenarios rather than population-wide screening.

DRE also maintains a role in clinical staging. Although mpMRI has improved local staging accuracy, DRE can still help identify extracapsular extension or locally advanced disease (≥T3), particularly when imaging findings are equivocal or discordant with clinical suspicion.

Importantly, the relevance of DRE is amplified in settings where access to high-quality mpMRI is limited. Global disparities in imaging availability and expertise mean that, in many regions, DRE remains a pragmatic and valuable component of the diagnostic pathway. In such contexts, its integration with PSA testing may still represent a reasonable and necessary approach.

While the repositioning of DRE reflects progress in prostate cancer diagnostics, it also highlights emerging challenges associated with increasing reliance on imaging. mpMRI, although highly valuable, is not without limitations. In addition to cost, variability in image acquisition, interpretation, and reporting continues to affect diagnostic performance and reproducibility. Interobserver variability among radiologists, differences in adherence to PI-RADS standards, and inconsistencies in training and quality assurance all contribute to heterogeneity in clinical outcomes (10).

Addressing these challenges requires robust standardization efforts. Structured training programs, certification processes, and quality control frameworks are essential to ensuring mpMRI delivers consistent, reliable results across institutions. Consensus initiatives, such as those led by the European Society of Urogenital Radiology, provide important guidance, but real-world implementation remains uneven (11). Without such standardization, the benefits of advanced imaging may be undermined, potentially reintroducing diagnostic uncertainty.

In this evolving landscape, the role of DRE should be understood within a broader framework of multimodal, patient-centered care. Rather than being abandoned, it should be selectively integrated into clinical pathways where it adds incremental value. This approach aligns with the fundamental principle of precision medicine: applying the right tool to the right patient at the right time.

In conclusion, DRE in 2026 is no longer a frontline screening modality but a complementary clinical instrument with specific indications. Its strengths lie in its accessibility, specificity when abnormal, and utility in selected diagnostic and staging contexts. As prostate cancer detection continues to evolve, the challenge is not to replace traditional tools outright but to refine their use within increasingly sophisticated diagnostic algorithms. The future of prostate cancer care will depend on the thoughtful integration of clinical examination, biomarkers, and advanced imaging to optimize outcomes while minimizing harm.

## Data Availability

Not Use Data.
